# Stereotactic Radiation for Oligometastatic and Oligoprogressive Stage IV Breast Cancer: A Case-based Review

**DOI:** 10.3390/curroncol30020192

**Published:** 2023-02-18

**Authors:** Gary M. Freedman, Joshua A. Jones, Neil K. Taunk

**Affiliations:** Department of Radiation Oncology, Perelman School of Medicine of the University of Pennsylvania, Philadelphia, PA 19104, USA

**Keywords:** stereotactic body radiation therapy, oligometastases, oligoprogression, metastatic breast cancer

## Abstract

For decades, the distant progression of breast cancer has been the purview of systemic therapy alone or with low to moderate-dose radiation therapy intended for the palliation of symptomatic metastases. However, for decades there have been anecdotes of long-term disease-free survival with more aggressive local treatment of one or more metastases. The hypothesis of oligometastases is that the treatment of a clinically limited number of distant metastases can change the natural history of stage IV breast cancer. The advance in the technology of stereotactic body radiation (SBRT) has made it more possible to offer a non-invasive, yet potentially disease-modifying, metastases-directed ablative treatment in place of surgery or a palliative radiation regimen. Although there are promising local control and survival outcomes in phase I/II trials, there is still a lack of phase III evidence of ablative SBRT results showing any change in the natural history of metastatic breast cancer. Limited oligometastases may call for an ablative approach with SBRT when definitive long-term local control is needed for the best palliation against symptomatic progression in challenging locations. Some oligometastases that have progression on a certain systemic regimen, while others remain stable or in remission, may also be treated with SBRT in the hopes of prolonging the use of that regimen. Whether SBRT should represent the standard management for stage IV breast cancer of a limited number or of limited progression requires confirmation by phase III data. This review will discuss the data from key clinical trials as it applies to decision making in typical clinical cases considered for potentially ablative SBRT for oligometastases or oligoprogression.

## 1. Introduction

The systemic hypothesis by Fisher had been the dominant model explaining the natural history of breast cancer and stage IV disease for 30 years, from approximately 1985–2015 [1]. This proposed that the natural history between localized breast cancer at presentation and metastases in distant organs was essentially, for the clinician, a simultaneous relationship. Even apparently early-stage breast cancer at diagnosis had widespread microscopic distant dissemination, even if not clinically apparent, by the available means of staging. For this reason, variations of initial local or regional treatment would not have a significant impact on the appearance of the systemic disease and survival.

This systemic model of breast cancer was prospectively validated by the phase III twin studies: NSABP B04, testing regional treatments in the mastectomy setting, and NSABP B06, testing local treatments in the lumpectomy setting [2,3]. In both trials, three different local-regional treatments intended to be good, better, or best effective at eradicating the local-regional disease resulted in the same distant disease survival and overall survival. This was the apparent proof that breast cancer was a disseminated disease even at presentation. A downstream result of this model was that only a systemic treatment could be used to treat systemic disseminated disease, and localized treatments such as surgery or radiation would have no role in managing stage IV breast cancer, other than when local control or palliation were needed.

A spectrum model proposed by Hellman suggested that some percentage of breast cancers may be detectable when of an only local-regionally limited extent before they have progressed to distant metastases [4]. In those hypothetical cases that are local-regionally confined, the eradication of local-regional disease could interrupt the later progression to distant metastases and result in a measurable improvement in survival. This hypothesis was prospectively validated by the meta-analysis of the phase III trials of postmastectomy radiation by the Early Breast Cancer Trialists’ Collaborative Group [5]. In patients with node positive breast cancer, postmastectomy radiation reduced both local-regional recurrences and distant metastases, resulting in improved breast cancer survival. In addition, phase III trials have shown small improvements in disease-free survival with elective regional node irradiation in women at higher risk for occult regional metastases to the internal mammary or supraclavicular regions [6,7]. Since 2015, the acceptance of this spectrum model of breast cancer spread has led to the increased incorporation into guidelines of postmastectomy radiation and elective regional node irradiation for most women with node positive breast cancer.

In a sense, limited regional nodal disease can be considered as the ‘original’ model of a state of limited, but potentially curable, metastases in breast cancer. The eradication of this local-regional disease, by radiation with or without effective systemic therapy, could change the natural history of the breast cancer, but does this model, when taken to a greater extreme, suggest that something similar may be possible in patients discovered to have a clinically limited number or isolated distant sites of stage IV disease—a condition known as oligometastases?

## 2. A Model of Oligometastases in Breast Cancer

**Figure 1:**
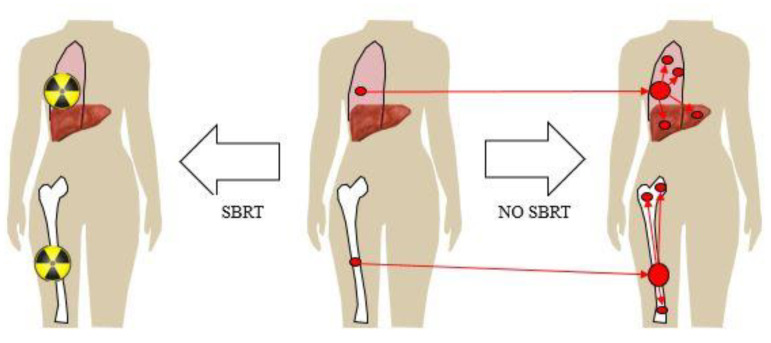
A model of oligometastases and metastasis-directed therapy. The center figure is of a woman with oligometastases in 2 known lesions, one in the lung and one in the bone. For the figure to the right, with standard management by observation or palliative therapy only, there can be both local progression of each of the known metastases, and secondary seeding to other foci of distant metastases. For the figure to the left, ablative metastasis-directed therapy, in this case stereotactic radiation therapy (SBRT), has resulted in ablation of the 2 known metastases and absence of further disease progression in the same or other organs.

Hellman and Weichselbaum first proposed the existence of an oligometastatic state, where a patient with breast cancer with a limited number of metastases could have improved long-term disease control with metastasis-specific treatment rather than systemic therapy alone [8]. Figure 1 illustrates one model of oligometastases and metastasis-directed therapy where there could be a benefit in reducing both the progression of the treated metastasis, and also the prevention of the secondary seeding of new metastases. In this way, improved treatment, or even the ablation of a metastasis, could change the natural history and improve survival in stage IV breast cancer. The theory behind a survival benefit to the treatment of oligometastases would depend on several conditions: compared to systemic therapy or palliative radiation alone, a more definitive local control achieved by aggressive therapy (surgery or radiation) would need to prevent death from the local progression of the known metastasis or prevent other subsequent metastatic disease that would lead to death, and not cause death itself from the intervention.

This model of oligometastases is in stark contrast to the current existing model that Stage IV breast cancer is always a widely disseminated disease, so that systemic therapy alone is the mainstay of management for slowing systemic progression and death from the disease. Currently, whether in the setting of oligometastases (limited) or polymetastases (extensive), the primary goal of metastasis-directed therapy is for palliation, whether by radiation, surgery or another local intervention, to provide relief from symptoms for the patient. Historically, radiation for distant metastases has been for the palliation of pain, bleeding, neurologic symptoms, risk of fracture, or other symptomatic local effects caused by the metastasis. The goal is to provide relief for as long as possible, preferably for the remainder of the patient’s life, and not necessarily ablation at the microscopic level of the metastasis itself. In this model, the metastases themselves do not contribute to the additional propagation of systemic metastases—they originate only from the primary itself.

## 3. Local-Regional Treatment in Stage IV Breast Cancer

**Figure 2:**
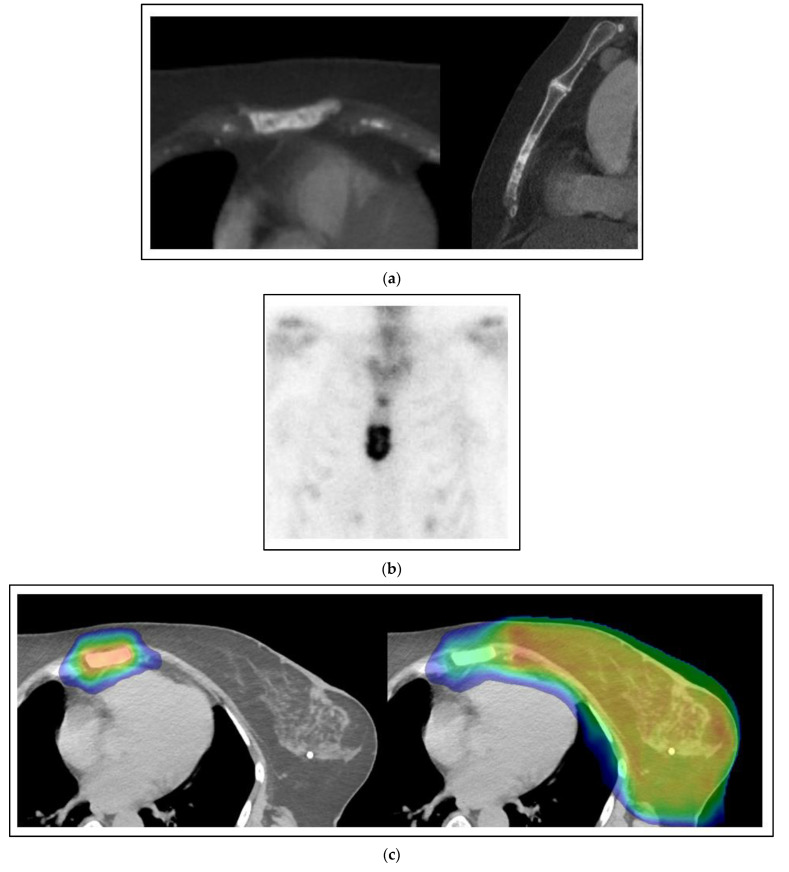

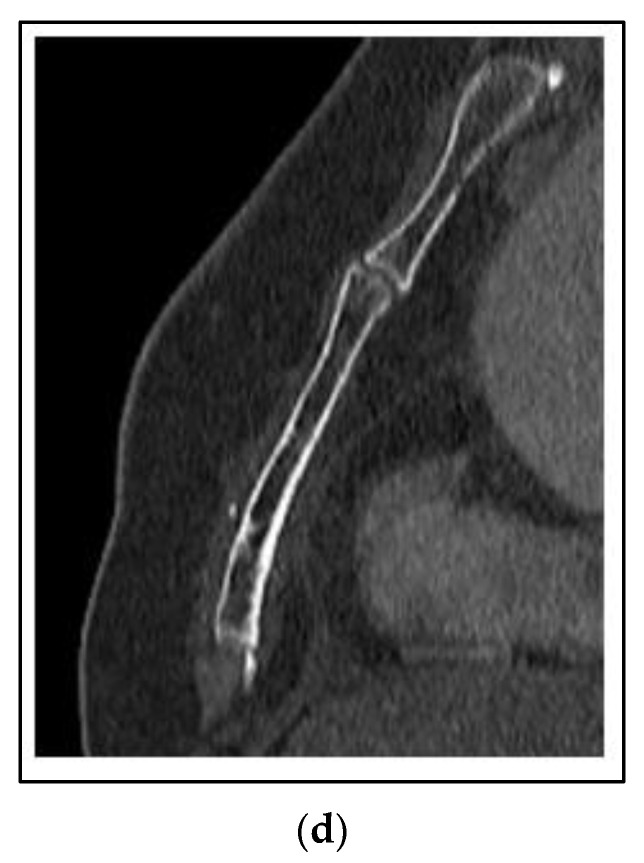
Case 1: Simultaneous oligometastases. A 60-year-old woman presents with a locally advanced cancer of the left breast cT1N3M1 with an oligometastasis of the sternum. The sternal oligometastasis is shown on representative images from CT (**a**) and bone scan (**b**) imaging. (**c**) Radiation therapy to the left breast and sternum. The left image shows a colorwash image superimposed on a radiation planning CT of the dose from the sternum-directed SBRT given 40 Gy in 5 fractions, and the right image shows the colorwash of the dose of 50 Gy given to the breast and regional nodes combined with the sternal treatment. The yellow color indicates the prescribed dose of radiation, and the green and blue colors around the edges indicate the medium and lower dose fall-off of the radiation dose plan (**d**) The appearance on a CT scan image of the sternum 3 years after treatment. There is minimal residual sclerosis of the sternum with no evidence of progression.

Figure 2 shows a case of a 60-year-old woman with the simultaneous presentation of left breast cancer involving the axillary and supraclavicular nodes with an oligometastasis of the sternum evident at the time of diagnosis. A biopsy of the axilla was positive for a grade 3 invasive carcinoma that was ER positive, PR positive and HER2 negative. Her staging for distant metastases was notable for a sclerotic lesion of the sternum on a CT that was positive on a bone scan. After neoadjuvant chemotherapy, an axillary dissection showed 2/14 nodes positive with pleomorphic invasive lobular carcinoma and extranodal extension. She was treated with comprehensive radiation to the left breast and regional nodes to a dose of 50 Gy with a boost to 66 Gy to the initially positive but undissected axillary and supraclavicular regions using external beam radiation. She concurrently had SBRT to the sternum 8 Gy × 5 fractions to 40 Gy. After radiation, she was treated with an aromatase inhibitor, cyclin-dependent kinase (CDK) 4 and 6 inhibitor, and zoledronic acid. She is without evidence of progression three years later.

In this case, the patient was treated with local-regional surgery and radiation in essentially the same fashion as for stage III breast cancer, despite the oligometastasis of the sternum. In a strictly palliative approach to stage IV breast cancer, where the woman is considered incurable, an argument could be made for an alternative management by systemic therapy alone. The woman is ER positive, PR positive and HER2 negative, with at least one known bone metastasis, so she could have been given first-line therapy with an aromatase inhibitor and CDK 4/6 inhibitor [9].

In several phase III trials of stage IV breast cancer, initial local-regional treatment has not been associated with a consistent overall survival benefit, but there have been improvements in local-regional control. In one phase III trial for patients with stage IV breast cancer, 716 patients were registered and a subgroup of 350 were randomized to local-regional treatment or systemic therapy alone [10]. Registered patients were eligible for randomization if they had an estimated life expectancy of at least six months, were fit to undergo general anesthesia for major surgery, had no local or distant progression or stable disease in response to the preceding chemotherapy, or ulceration, fungation or bleeding at the local site that mandated palliative locoregional treatment. Surgery was followed by standard postoperative adjuvant radiation treatment to the chest wall or remaining breast as per the standard institutional practice for non-metastatic patients. Only 25% of the patients had oligometastases defined as ≤3 metastases. There were no differences in the median or two year survival, including in the subgroup analysis of oligo versus polymetastases. In another phase III trial of surgery or no surgery at stage IV presentation, where 38% of the patients also had postmastectomy radiation therapy, there was a trend for survival improvement with better local-regional control [11]. The median survival period after radiation was 41 months versus 35 months for those without radiation (*p* = 0.36). The hazard of death was 34% lower in the local-regionally treated group and, at 5 years, 42% of the patients were alive versus 24% (*p* = 0.005). Younger age, ER/PR positive, and solitary bone only metastases were associated with better overall survival. The ECOG-ACRIN Research Group conducted a randomized phase III trial of systemic therapy plus early local therapy versus systemic therapy alone in women with stage IV breast cancer [12]. In total, 390 patients with stage IV breast cancer and an intact primary breast tumor were enrolled. If there was no progression after 4–8 months of systemic therapy, 256 eligible patients were then randomized to continued systemic therapy alone (131 patients) or with loco-regional surgery and radiation (125 patients). There was no significant difference in the three year overall survival (OS), progression-free survival (PFS) or health-related quality of life. However, local-regional treatment was more closely associated with lower local-regional recurrence/progression than systemic therapy alone (10.2% vs. 25.6%, *p* = 0.003). The initial reported abstract did not contain separate analysis for oligo- versus polymetastases.

In summary, for this patient with stage IV breast cancer with an expectation of prolonged life expectancy on modern systemic therapy, there is better local-regional control with surgery ± radiation. In this case, with extensive regional disease of the axilla and supraclavicular region, the patient was at high risk >30% for experiencing a local-regional recurrence with surgery alone. Local therapies of the breast, including radiation, may create the conditions for minimizing the risk of relapse and progression that could cause pain, symptoms, skin wound, infection, or a constellation of all the above that reduces quality of life. A regional relapse or progression, particularly with supraclavicular disease, can cause symptoms including pain, lymphedema, venous thrombosis, neuropathy or brachial plexopathy, or skin wounds. The goals for her aggressive surgery and radiation were durable local-regional control and prophylactic palliation. However, any effects on her distant disease-free progression and overall survival are not uncertain from the existing phase III data.

## 4. Metastasis-Directed Therapy for Simultaneous Oligometastases in Stage IV Breast Cancer

The treatment of this patient’s sternal oligometastasis can be for palliation or more durable local control. The patient in Figure 2 could well have been recommended observation alone if the sternum was asymptomatic, or a palliative radiation dose of 8 Gy in 1 fraction, 20 Gy in 5 fractions or 30 Gy in 10 fractions for symptom control alone. Instead, she was treated with SBRT to the sternum 8 Gy × 5 fractions to 40 Gy. Can there be a survival benefit to ablative-intent therapy of this oligometastasis by SBRT?

The Stereotactic Ablative Radiotherapy for the Comprehensive Treatment of Oligometastases (SABR-COMET) trial was a randomized phase II for patients with a controlled primary tumor for at least three months and between one and five metastases that were deemed amenable to SBRT in multiple different cancer types, including breast cancer (Table 1) [13]. The control arm was standard systemic therapy alone or with palliative radiation of 8 Gy in 1 fraction to 30 Gy in 10 fractions, at the discretion of the treating physician. The investigational arm was standard systemic therapy with SBRT to all the oligometastases. The primary site was breast cancer in only 18 of 99 patients—5/33 in the control arm and 13/66 in the SBRT arm. The eight year overall survival of all patients was 27% vs. 14% (*p* = 0.008) and progression-free survival was 21% vs. 0% (*p* < 0.001). There was no difference in the time to new metastases overall or the development of bone, brain, liver, or lung metastases. Only two of the original 18 breast patients, both in the SBRT arm, were alive without progression for more than five years. There was greater toxicity with SBRT grade ≥ 2 30% vs. 9% *p* = 0.019) and three deaths in the SBRT arm (4.5%). Another exploratory analysis revealed a benefit seen with SBRT in a lower use of cytotoxic chemotherapy after SBRT (33% vs. 55%, *p* = 0.043) and a lower use of palliative radiation after SBRT (24% vs. 70%, *p* < 0.001).

Based on this randomized phase II data, SBRT to the oligometastasis in her sternal lesion offered the hope for improved progression-free and overall survival compared to systemic therapy with or without palliative radiation alone. One in five patients may be alive without progression or new metastases >5 years. In addition, SBRT offers improved durable long-term control, if not out-right total ablation, of the oligometastasis compared to only the observation or palliative treatment of it. The phase III data are pending completion and reporting of the follow-up SABR-COMET-3 and SABR-COMET-10 studies [17,18].

**Figure 3:**
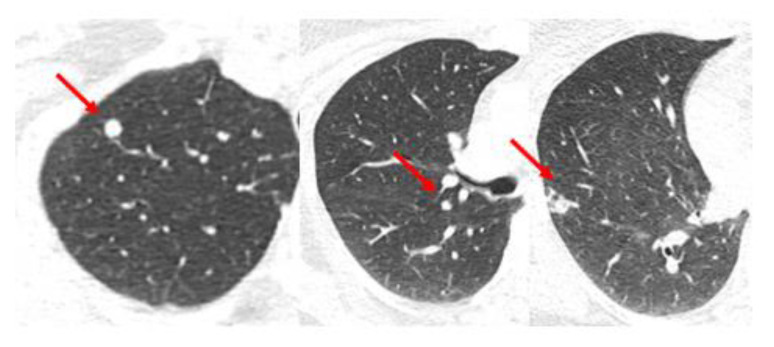
Case 2: Simultaneous oligometastases. Three representative axial images from a CT scan obtained for initial staging of breast cancer showed 3 highly suspicious lung nodules each marked with a red arrow that were PET-avid. With systemic therapy alone there has been a complete response in the lung nodules for greater than 3 years.

In the previous case, there was not a complete response to neoadjuvant chemotherapy in the local-regional disease or in the sternum. However, what is the role of SBRT if an oligometastasis is more responsive to systemic therapy? Figure 3 shows images of a CT scan of the chest conducted for the staging of breast cancer showing three suspicious lung nodules at diagnosis that were PET-avid. The woman is a 68-year-old who presented with right breast cancer T3 N1 M1, with no other signs of metastatic disease. She had neoadjuvant chemotherapy with TCHP. She had a clinical complete local-regional response. She then had lumpectomy and sentinel node biopsy, followed by local-regional radiation. Her first three month restaging CT showed a partial response in the lung nodules, and by the six month CT scan on HP alone, there was a complete resolution of the lung nodules. She continues on HP over three years from initial treatment and is without signs of recurrence or progressive disease.

There are data to suggest that the observation of these oligometastases was the best approach in this woman, with a good response to systemic therapy. The NRG BR002 trial was a randomized phase II trial exclusively for breast cancer patients with 1–4 extracranial metastases, controlled local-regional disease, and no clinical progression for up to 12 months on systemic therapy (Table 1) [14]. Overall, 125 patients were randomized to standard systemic therapy (*n* = 65) versus systemic therapy plus metastasis-directed therapy (*n* = 60). The oligometastases in the 60 patients assigned to the ablative treatment arm received SBRT in 56 patients (93%), surgery in 1 patient (2%), and no protocol therapy in 3 (5%). Furthermore, 78% had metachronous and 22% had synchronous oligometastases. After a median follow-up of 35 months, the mean progression-free survival (PFS) was 23 months versus 19.5 months, and 3-year PFS 33% versus 38% (*p* = 0.36). The 3-year OS was 72% versus 69% (*p* = 0.54). There was no difference in the appearance of new metastases outside the treated oligometastases (40%), but there was a lower rate of progression within the oligometastases treated by SBRT (7% versus 29%). There was a high quality assurance and pre-review of the treatment plans and no grade 5 toxicity. Based on the phase II analysis, the subsequent planned phase 3 trial enrollment was cancelled.

Therefore, in this patient with lung metastases responding well to systemic therapy, observation without SBRT directed to each of the three lung metastases was appropriate. The patient was spared any risk for toxicity from surgery or SBRT, and the results of the NRG BR002 do not support a hypothesis that metastasis-directed therapy could reduce the incidence of other distant metastases. The three oligometastases remain in remission, but if there was a detectable local recurrence in one or more of them by CT surveillance, then SBRT for local control could be considered in the future.

## 5. Metastasis-Directed Therapy for Metachronous Oligometastases in Stage IV Breast Cancer

**Figure 4:**
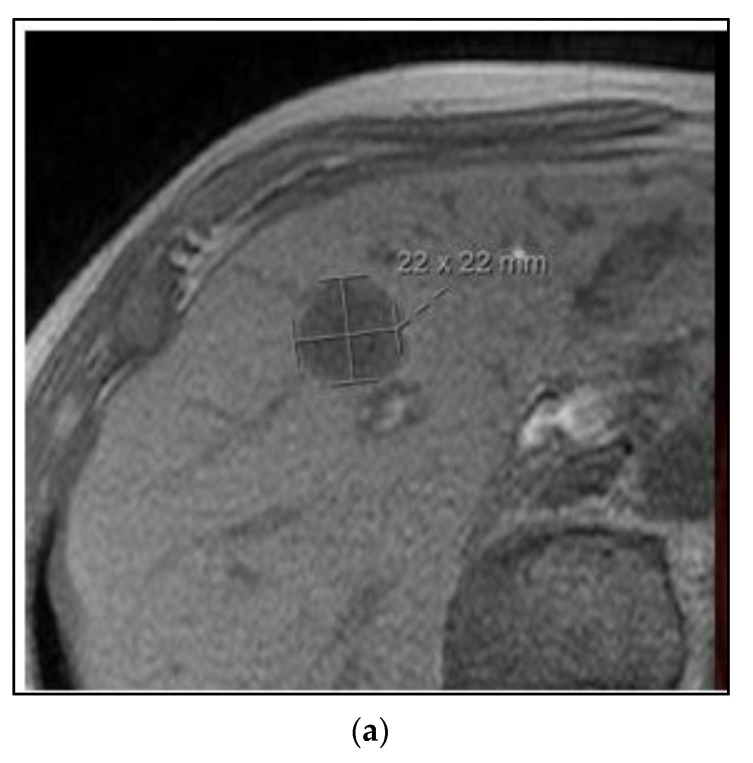

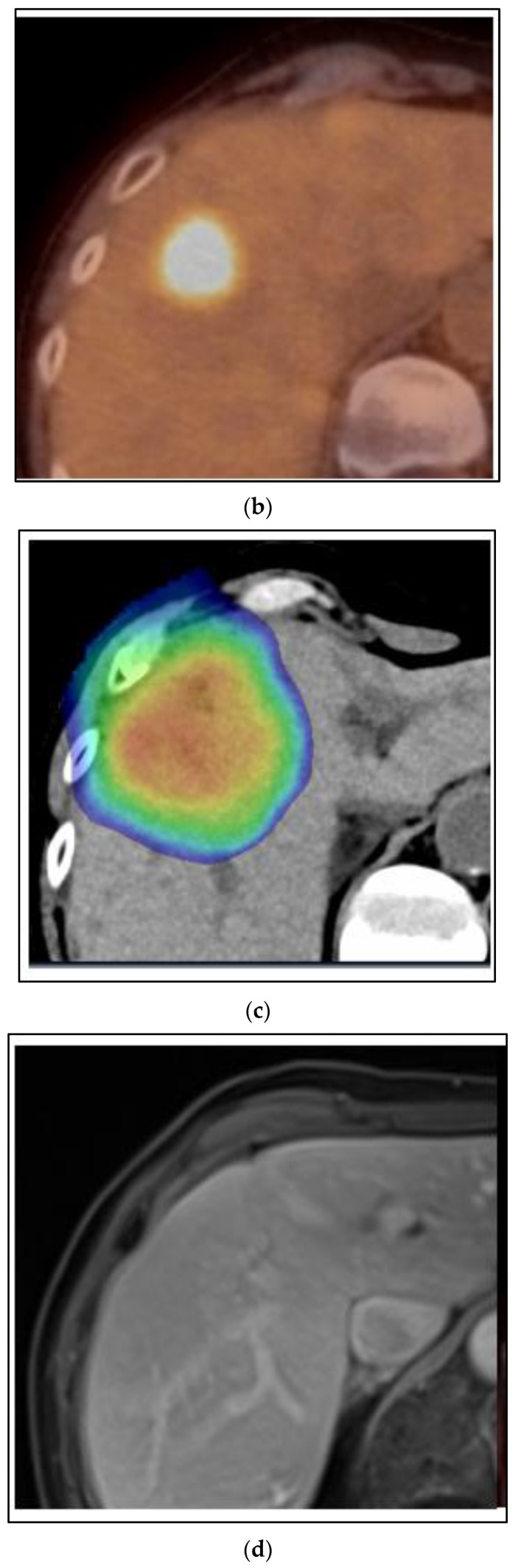

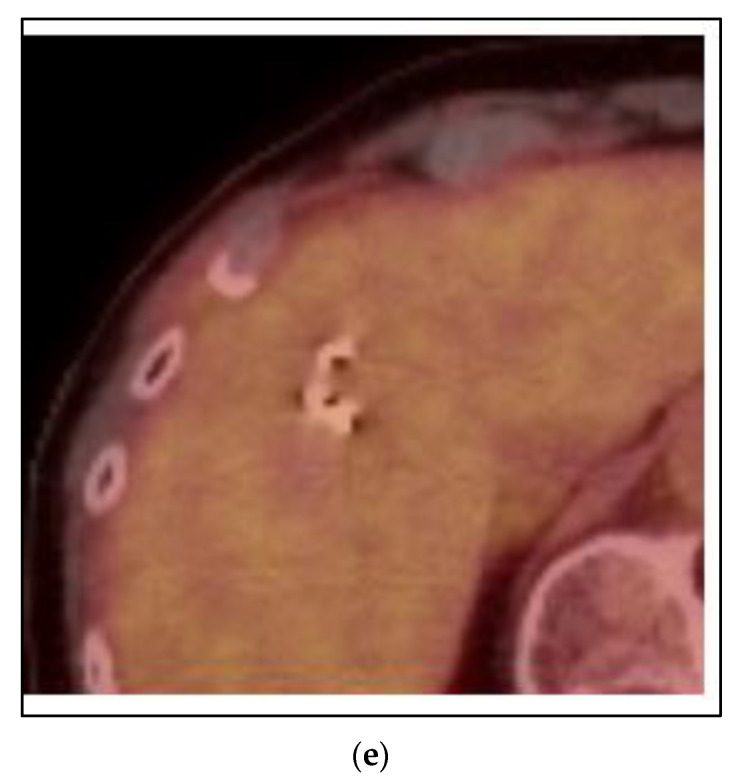
Case 3: Metachronous oligometastases. A 75-year-old woman presented with metachronous metastatic breast cancer to the liver over 5 years from her initial treatment. She was treated with SBRT to both liver lesions. MRI (**a**) and PET/CT (**b**) imaging of one of the two liver metastases. (**c**) A colorwash image superimposed on a radiation planning CT showing the dose from SBRT to 50 Gy in 5 fractions using proton beam radiation. Representative images showing a complete response on MRI (**d**) and PET/CT (**e**) 4 years after SBRT treatment.

The majority of the patients in the BR002 trial had metachronous oligometastases after the initial local-regional treatment. Figure 4 shows a case of a 75-year-old woman with a metachronous presentation of oligometastatic breast cancer to the liver. Her initial node positive breast cancer was ER positive, PR positive, and HER2 negative and treated more than five years earlier with mastectomy, postmastectomy radiation, chemotherapy and five years of tamoxifen. She developed abdominal symptoms and elevated liver function tests and imaging showed two lesions of the liver, 2.2 cm and 0.8 cm. A liver biopsy was positive for metastatic breast cancer of the same receptor pattern as the original primary. She was treated with SBRT to both liver lesions, followed by an aromatase inhibitor and remains without evidence of disease progression four years later.

In this case, the two oligometastatic lesions in the liver were metachronous from the primary breast cancer and occurred on her first-line adjuvant endocrine therapy. One option for management could have been a change in her systemic therapy alone, from tamoxifen to an aromatase inhibitor with a CDK 4/6 inhibitor. The data from the SABR-COMET and NRG BR002 phase II trials are contradictory in terms of whether there would be an improved PFS and OS with SBRT. The National Health Service England conducted a large prospective registry of SBRT for metachronous 1–3 oligometastases (Table 1) [15]. Patients were eligible for enrollment with 1–3 oligometastases and a disease-free interval from the primary cancer to metastases >6 months. Among 1422 patients, only 78 (5.5%) had primary breast cancer. In this subgroup, the local control of the metastases was 78% at two years and the two year OS was 83%. There was no grade 5 toxicity. In this NHS study, the progression of disease was in fact the most common cause of death. Therefore, it could be expected that the local control of the liver lesions would be better with SBRT than palliative radiation alone. Failure to achieve liver control is a major potential cause for morbidity and mortality in the future, and based on the SABR-COMET, there could also be a lower risk for needing cytotoxic chemotherapy in the future; this is potentially important in the case of this woman, aged 75.

## 6. SBRT for Oligoprogression of Stage IV Breast Cancer

**Figure 5:**
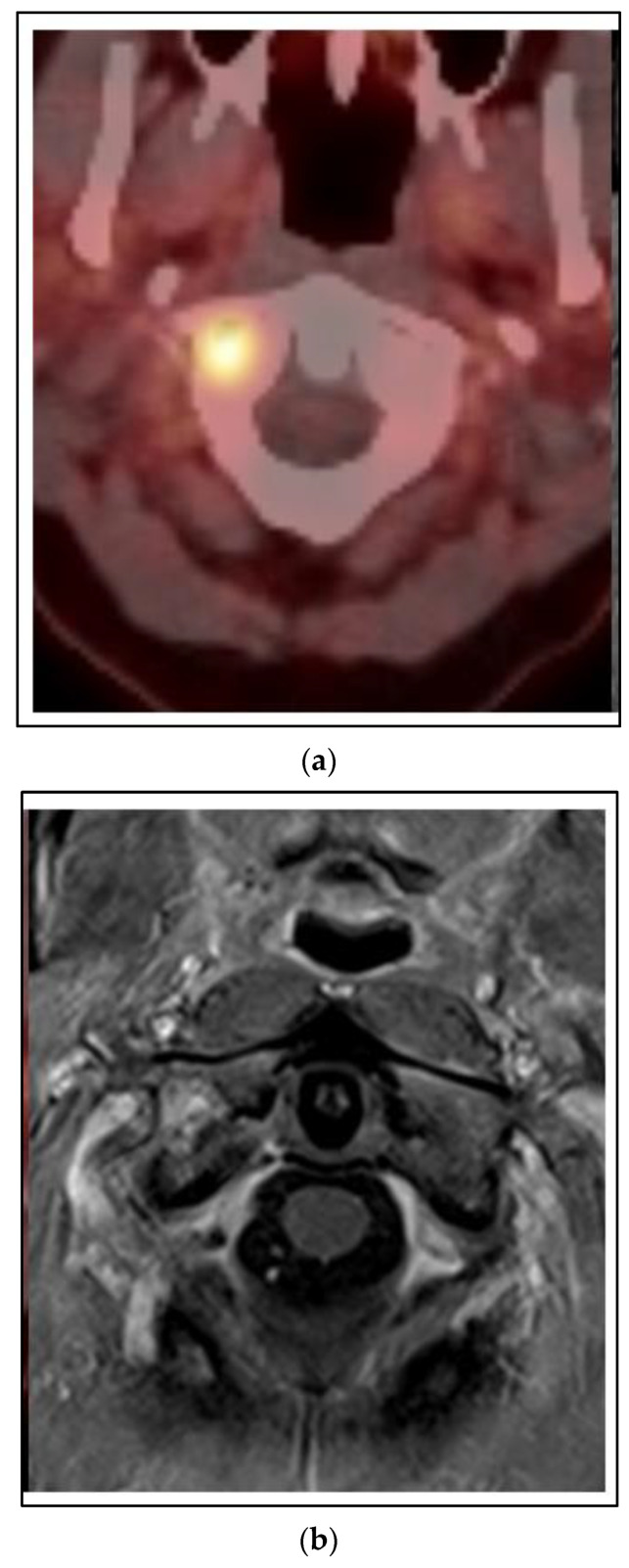

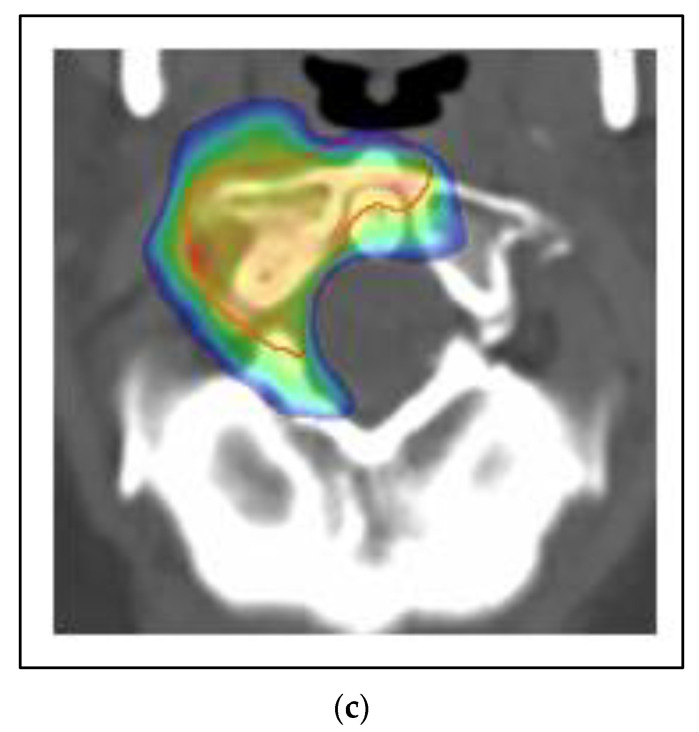
Case 4: Oligoprogression in the C1 vertebra. A 45-year-old woman presented with oligoprogression at C1 on systemic therapy for metachronous bone metastases. Representative images of the C1 metastasis on PET/CT (**a**) and MRI (**b**). (**c**) A colorwash image superimposed on a radiation planning CT showing the dose from SBRT to the C1 vertebral metastasis 24 Gy in 2 fractions with photon IMRT.

Cases 1–3 have been of synchronous or metachronous oligometastases. In a case of oligoprogression, there can be one or more limited number of sites of metastases that have not responded or progressed on a systemic regimen, while the others remain under control. Figure 5 shows a case of a 45 year old woman who presented with left breast cancer, clinical T1c N1 M0, ER+/PR+/HER2−, stage IB. She had neoadjuvant chemotherapy and bilateral mastectomy with flap reconstruction showing residual disease in the breast and axilla T1c (3) N2a. She was treated with adjuvant tamoxifen. After two years, she developed bone pain, and a bone scan was positive for an extensive number of metastases of the neck, ribs and spine. She was changed to leuprolide, aromatase inhibitor and CDK 4/6 inhibitor. However, within one year, she developed neck pain and an MRI and PET/CT showed a progression of the C1 vertebra. Her other disease was stable. She was treated with SBRT 24 Gy in 2 fractions. She had complete relief of her neck pain and continued on the same systemic therapy.

In this case of extensive polymetastases, one option for the management of stage IV breast cancer that progressed on systemic therapy would be to change the systemic therapy. The Consolidative Use of Radiotherapy to Block oligoprogression (CURB) trial randomized patients with breast or lung cancer with up to five extracranial sites to the standard of care systemic therapy and SBRT up-front or delayed at further progression (Table 1) [16]. Overall, 106 patients were reported with a median follow-up of 45 weeks. There was an improvement in the PFS for SBRT to oligoprogressive foci for the lung cancer cohort (median 44 weeks vs. 9 weeks, *p* = 0.001), but not the breast cancer cohort (18 weeks vs. 19 weeks, *p* = 0.478). Based on these data, SBRT may not be associated with the change in the natural history of her metastatic breast cancer.

However, the option of SBRT to this one area of oligoprogression in the cervical spine could allow her to remain on her current, well-tolerated systemic therapy, which is controlling her other polymetastases, and avoid a change to cytotoxic systemic therapy. More importantly, the consequences of local progression in this location of the cervical spine are potentially severe. Failure to achieve local control at C1 could cause severe morbidity from fracture or surgery, immobilization, a decline in performance status, paralysis or even death. In this case, local control is essential to preserving not only quality of life, but survival itself.

The SC24 phase II/III trial randomized patients with painful spinal metastases to SBRT or moderate-dose palliative external beam radiation [19]. Treatment with SBRT 24 Gy in 2 fractions was compared to conventional palliative external beam radiation 20 Gy in 5 fractions. In total, 229 patients were enrolled and randomly assigned to receive conventional external beam radiotherapy (*n* = 115) or stereotactic body radiotherapy (*n* = 114). At three months, 40 (35%) of the 114 patients in the stereotactic body radiotherapy group, and 16 (14%) of the 115 patients in the conventional external beam radiotherapy group had a complete response for pain (*p* = 0·0002). With a longer follow-up in a subgroup of 137 patients, the risk for local failure was 15% for SBRT vs. 36% for palliative EBRT at two years (*p* < 0.001) [20]. The need for re-irradiation at one year was 2% for SBRT and 16% for palliative EBRT (*p*-0.002). There were grade 3 vertebral compression fractures associated with SBRT in five patients (8%), but this was comparable to the rate of fractures from the progression of disease after conventional palliative radiation.

Therefore, for this case of oligoprogression in a critical cervical vertebral location, SBRT offers the best long-term palliation at C1 by offering the best local control compared to low-moderate dose palliative external beam radiation. Whether SBRT, in cases of breast cancer oligoprogression such as this, alters the natural history of PFS elsewhere in other metastatic locations or changes the OS is unproven in the phase III data, but is of less concern than having durable local control in this cervical spine location.

## 7. Conclusions

This review of SBRT for oligometastases and oligoprogression in stage IV breast cancer discussed several key clinical trials used for medical decision making in the four case examples presented (Table 1). Additional data is forthcoming in several additional phase III trials. The Superiority of Stereotactic Body Radiation Therapy in Patients With Breast Cancer (STEREO-SEIN) trial (ClinicalTrials.gov Identifier: NCT02089100) is a phase III trial, enrolling patients with hormone-sensitive breast cancer and randomizing them to systemic therapy and palliative metastasis directed therapy versus systemic therapy and SBRT to all metastases. The Metastases-directed Radiotherapy in Addition to Standard Systemic Therapy in Patients With Oligometastatic Breast Cancer (OLIGOMA) trial (ClinicalTrials.gov Identifier: NCT04495309) is preferably ablative radiation with few high-dose fractions; however, 3D or IMRT in moderate or standard fractionation can be used for larger metastases or metastases near critical organs. The Conventional Care Versus Radioablation (Stereotactic Body Radiotherapy) for Extracranial Oligometastases (CORE) trial (ClinicalTrials.gov Identifier: NCT02759783) is a phase II/III trial that includes breast, prostate and lung cancer.

For decades, the treatment of high-risk local-regional disease had the same uncertain status in cases of node positive or locally advanced breast cancer. In inflammatory breast cancer, with a survival benefit or not, PMRT is necessary for the prevention of morbid and even life-threatening local-regional progression. In a similar way, even if SBRT is “just” for better local control than standard external beam palliative radiation, for many patients, this more effective local control may actually be the most effective palliation and mitigates the risk of re-irradiation or other therapy for local control. The effective palliation of metastases in critical locations such as the spine, weight-bearing appendicular bones or critical organs can be associated with morbidity and mortality if not effectively treated by radiation, particularly in the setting of progression on systemic therapy. Clinical judgement is needed to determine when the greater local control benefit of SBRT is outweighed by the low risk for toxicity from SBRT. Whether a survival benefit to metastasis-directed SBRT in stage IV breast cancer exists remains to be determined by more phase III trials in breast-specific patient populations.

## Figures and Tables

**Table 1:** Key clinical trials referred to in this review testing SBRT for oligometastases or oligoprogression in Stage IV breast cancer.

Trial	Phase	Number of Metastases	Number Patients	Number Breast Patients	Reference
SABR-COMET	IIR	1–5	99	18	[13]
NRG-BR002	IIR	1–4	125	125	[14]
NHS England	II	1–3	1422	78	[15]
CURB	III	1–5	106	47	[16]
